# The state of ethics education at medical schools in Turkey: taking stock and looking forward

**DOI:** 10.1186/s12909-020-02058-9

**Published:** 2020-05-24

**Authors:** Mustafa Volkan Kavas, Yesim Isil Ulman, Figen Demir, Fatih Artvinli, Melike Şahiner, Meral Demirören, Gamze Şenyürek, Işıl Pakiş, Nadi Bakırcı

**Affiliations:** 1 Department of History of Medicine and Ethics, Ankara University, School of Medicine, Morfoloji Building, 06230 Ankara, Altındağ Turkey; 2 Department of History of Medicine and Ethics, Acibadem University, School of Medicine, Kayışdağı Caddesi No:32, 34752 İstanbul, Ataşehir Turkey; 3 Department of Public Health, Acibadem University, School of Medicine, Kayışdağı Caddesi No:32, 34752 İstanbul, Ataşehir Turkey; 4 Department of Medical Education, Acibadem University, School of Medicine, Kayışdağı Caddesi No:32, 34752 İstanbul, Ataşehir Turkey; 5 Department of Medical Education and Informatics, Hacettepe University, School of Medicine, 06230 Ankara, Altındağ Turkey; 6 Department of Forensic Medicine, Acibadem University, School of Medicine, Kayışdağı Caddesi No:32, 34752 İstanbul, Ataşehir Turkey

**Keywords:** Inventory, survey, ethics curriculum, workforce, teaching and learning, assessment and evaluation, medical schools, Turkey

## Abstract

**Background:**

Ethics teaching is globally considered an essential part of medical education fostering professionalism. It does not only provide knowledge for good clinical conduct, but also trains medical students as virtuous practitioners. Although Turkey has had a considerable experience in ethics education of healthcare professionals, the general state of ethics curricula at medical schools in Turkey is unknown.

**Methods:**

The purpose of this study was to collect comprehensive data about the ethics education programs at medical schools in Turkey. To this aim, we designed a cross-sectional descriptive questionnaire survey which focuses on the content, teaching years, teaching, assessment and evaluation methodologies, workforce and infrastructure. We delivered the questionnaire to all medical schools in Turkey. Seventy-nine medical schools participated in this study (response rate: 78%).

**Results:**

Although most institutions had an undergraduate ethics curriculum (91.1%), the findings suggest deficiency of teaching personnel (34.2% had no instructors). Furthermore, the distribution and composition of the workforce was imbalanced. The content varies largely among institutions. Medical schools with an ethics department were more likely to diversify teaching topics. However, ethics education was largely based on the four-principle approach. The content was usually conveyed to students theoretically. Around 90% of schools had classroom lectures. It is the only method used at one-third of them. Clinical ethics education was mostly lacking. Multiple-choice tests were widely used to assess and evaluate student attainments (86.1%).

**Conclusions:**

Staff qualified to teach ethics and ethics education integrated into the six-year medical curriculum given by a multidisciplinary team are urgent necessities. Considering teaching, assessment and evaluation methodologies used, most medical schools seem to fall short of fostering students to develop ethical attitudes. Endeavors aiming for modern topics should be encouraged. As the organization ethics education change continuously, we think that a platform for monitoring ethics education at medical schools in Turkey should be established. Such a body would help ethics instructors to network and find solutions to current problems and build shared wisdom.

## Background

In the last few decades, bioethics has come into prominence in health sciences curricula. This change can be attributed to factors such as: morally and ethically challenging developments in science and technology, significant societal and ideological changes, and an increase in public awareness and demand for responsible healthcare. As a consequence, globally ethics education is seen as crucial to medical training today. Although bioethics and medical ethics have been widely integrated into undergraduate medical education, the purpose, content, outcomes, evaluation and assessment, and teaching methods have been under scrutiny of researchers [1].

Describing medicine as an inherently moral profession, Pellegrino and Thomasma argue that the ultimate goal of medical ethics education is to create virtuous physicians. Accordingly, the goal of ethics education is to help students develop the ability to engage in self-criticism and examine their own values and approaches in the face of moral dilemmas [2]. However, Eckles et al. draw attention to more measurable objectives. They suggest teaching ethical reasoning skills to students so that they can detect and resolve moral dilemmas in specific situations [1]. Many argue that moral education aims for both: namely, cultivating virtuous physicians and providing them with practical tools (i.e., knowledge and skills) for competent critical reasoning [3, 4].

In recent years, remarkable efforts have been made worldwide to standardize the core goals and methods of education programs and training approaches. For instance, the United Nations Educational, Scientific, and Cultural Organization (UNESCO) Division of Ethics of Science and Technology included principles such as human dignity; respect for vulnerability and personal integrity; non-discrimination and non-stigmatization; solidarity and cooperation; and protection of the environment, biosphere, and biodiversity in its Bioethics Core Curriculum document [5]. The World Medical Association (WMA) recommended that teaching medical ethics and human rights should be obligatory in the undergraduate curriculum and medical schools should be equipped with sufficient number of faculty members who are skilled at teaching ethical enquiry [6]. The American Board of Internal Medicine has declared primacy of patient welfare, autonomy, and social justice as the fundamentals of medical practice and draws attention to their inclusion in medical education [7]. Similarly, the General Medical Council outlined a professionalism-based framework for ethics education of future physicians in *Tomorrow’s Doctors* [8]. Despite these initiatives, bioethics and medical ethics education programs differ greatly not only across countries but also across institutions within a country, in terms of content; teaching approaches and techniques; and evaluation and assessment methods [1, 9–14].

In Turkey, ethics education at the under-graduate and post-graduate level for healthcare students has been improving recently. While a limited number of medical schools launched ethics education programs in the 1980s, in the current age medical ethics and related topics are an important component of professional education at most of the medical schools and at nearly all highly-reputed nursing schools [15–19]. In keeping with developments in Western countries, many healthcare training and research institutions have introduced topics concerning professionalism, identity development, and professional codes of conduct in their education programs.

Although specific guidelines on bioethics and medical ethics education and professionalism in Turkey are largely absent, the importance of ethics education has been mentioned a few times in the National Core Undergraduate Medical Education Program developed in 2014 by a task force composed of representatives of medical schools [20]. This issue has also been a hot topic in national literature. For example, the Cambridge Consortium for Bioethics Education Turkey Working Group recently published a report about contemporary approaches, needs, and necessities for proper ethics education in health sciences [21]. Similarly, Vatansever (2012) recommends using interactive and practical teaching methods intertwined with theoretical sessions throughout a six-year spirally-integrated ethics education program at medical schools [22]. However, a thorough answer to whether any, or how many, of these recommendations were put into practice at medical schools in Turkey has not been revealed yet. Almost two decades have passed since the most comprehensive national report mapping medical ethics education was released [23]. In addition, the Turkish Bioethics Association organized four separate thematic symposia on ethics education in healthcare in 2004, 2012, 2013, and 2015 [24, 25]. Thus, currently, inferences regarding the status of bioethics and/or medical ethics education continue to depend largely on personal observations or on scanty, small-scale research, and are not reliable. The scope of recent studies concerning under-graduate and post-graduate ethics education is too limited or specific [26–33].

In conclusion, the general state of ethics education at medical schools in Turkey is unknown. In the last twenty years, Turkey has undergone structural transformations in the healthcare sector. The economic, societal, and moral consequences of such transformations are claimed to have a direct influence on professional healthcare education [34, 35]. Regretfully, during this period, no extensive data was produced about the structure of ethics education programs with regard to variables such as the type of institutions (public or foundation) and features of the places where medical schools are situated (population of cities, regions with economic disparity, etc.).

A comprehensive up-to-date report demonstrating where we are in terms of ethics education in the healthcare sciences is needed. Studies designed to meet this need would provide a knowledge base for endeavors that aim to improve ethics education programs to align with international standards. For example, by this means, best practices could be unveiled and introduced to a broader audience. The strengths and weaknesses of ethics education programs could also be determined, and thus urgent problems and needs could be identified clearly.

## Methods

### Aim

The main purpose of this study was to collect comprehensive data about the ethics education programs at medical schools in Turkey. Our research aimed to present an overview of the state of affairs while focusing on aspects including faculty composition, course topics, duration, and teaching, assessment and evaluation methodologies.

### Study type

This study is a cross-sectional descriptive survey on formal ethics education at medical schools in Turkey. Since the study considers all medical schools in Turkey to be equal, power analysis was not conducted.

### Development of the questionnaire

The data collection tool (Questionnaire of Inventory of Ethics Education Curricula at Medical Schools in Turkey) was developed by the researchers specifically for this study (Additional File 1). The questionnaire comprises three parts. The first part contains 9 items about demographic information. In the second part, there are questions on 7 parameters including the content of medical schools’ bioethics and/or medical ethics curricula, teaching and training, the assessment and evaluation methods they utilize, as well as the qualities of their substructures. The last part of the questionnaire consists of 2 open-ended questions through which the participants may express their opinions about the strongest aspects of their bioethics and/or medical ethics education programs, and areas for improvement.

Initially, we conducted a literature review to identify the main topics in contemporary bioethics and medical ethics. Among them, to begin with, we adopted the first volume of the UNESCO Bioethics Core Curriculum book (Syllabus Bioethics Education Program) as the basic framework [5]. Afterwards, we enriched this content by comparing the initial topics with those covered in the *Tomorrow’s Doctors* report [8], the US-based Accreditation Council for Graduate Medical Education's Outcome Project [36], and the National Core Curriculum developed and proposed by the Council of Medical Schools Deans as a guideline for medical educators in Turkey [20]. We designed the questionnaire so that it included items that not only asked about which topics were covered in the education programs of different institutions, but also how they were treated. Thus, for each topic the following aspects were also examined: duration devoted to each teaching activity, teaching methods utilized, who conducted the activities, and assessments and evaluations. Next, we included a section about the participating institutions’ substructures and workforce. Here we aimed to learn whether they had an ethics department and/or an associated institute active in the organization of education programs, the number of people engaged in teaching activities, whether they had formal bioethics or medical ethics expertise, and if not, their professional backgrounds and their academic positions and titles. Finally, we added a commentary section, as explained above.

### Data collection

We delivered the questionnaire to all medical schools in Turkey along with an informed request from the higher management of those institutions or faculty members who were responsible from the conduct of education programs. We delivered the questionnaire first to the scholars working in the departments of history of medicine and ethics^1^ (DHMEs). In case there were no such departments at a medical school, we contacted the faculty administrators and requested them to direct us to persons responsible for the organization and conduct of ethics education there. Occasionally, we asked members of the department of medical education to either fill out the questionnaire themselves or help us reach the appropriate persons. Mostly via multiple e-mails, we sent out a secure link to the questionnaire after converting it to a digital format that allowed online participation. We called unresponsive participants for reminders and, in rare cases, collected their data via telephone interview. One of the researchers had telephone conversations with two participants and transcribed their answers into the online questionnaire. We collected the data between April 2017 and August 2018.

### Statistical Analysis

In the analysis of the data, we used frequency (percentage) for qualitative variables, and calculation of mean value (±standard deviation) for quantitative variables using package software. We presented descriptive analyses using frequency tables for the categorical variables. The Chi-square and Fisher’s exact tests were used to compare categorical variables. A Mann-Whitney U test was used to compare student quotas per year from each medical school and the number of assessment and evaluation methods used.

### Qualitative Analysis

Answers to the open-ended questions were analyzed by using inductive content analysis [37] by one of the researchers, who is a medical education expert. The steps of the qualitative analysis are as follows. Each answer given to an open-ended question was copied and pasted to a line in Excel and reviewed thoroughly and iteratively (open coding). Every emphasized attribute in each line was named as a theme and entered into a column in order to better understand the strengths and problems mentioned by participants regarding their teaching programs (transcription on a coding sheet). All answers were overviewed twice with intervals. Emerging themes were grouped as particular categories according to their meaning-closeness (grouping the data and creating a pattern). Consideration of the weight of a particular theme in the whole open-ended data was mainly based on the thematic pattern. The frequencies of themes were also calculated and the results were referred as subsidiary findings in this phase. The descriptive data was interpreted by considering the whole text (re-contextualization). The thematic pattern of the responses and quotation examples are given in Table 1.

**Table 1:** Thematic pattern of the answers given to the open-ended questions and sample quotations

Open-ended questions	Themes	Sub-themes	# Responses	Sample quotations (N37, N48, N59, ... etc. denote the participant order in the raw data)
What are the **strong aspects** of the ethics education at your school?	Experienced teacher	− Working for longer years − Being experienced in teaching ethics − Being open to multidisciplinary activities	21	*N37_Our strengths are: Sufficient number of faculty members - faculty members are heterogeneous and wide ranging - and having a good medical history library.* *N48_Ethics education is provided by a medical history-ethics doctorate who had previous experience in ethics and experience in medicine as well.*
	Educational model	− Diversity of course topics − Being supervised by national bodies − Using sources in English − Using a variety of teaching methods − Interactive sessions	16	*N59_Our education model is efficient because of the active participation of students in diverse educational activities such as small group works, case discussions, and preparing term papers besides amphitheater presentations.*
	Vertical/clinical integration	− Formal ethics education in clinical years − Ethics education during internship	7	*N17_ ... the ethics education of undergraduate students starts in the first year and takes place in the curriculum in different intensities in every educational phase.* *N25_In the integrated medical education curriculum of our faculty, in the first semester, medical ethics courses are currently given as a whole in the course program named "Evidence Based Medicine and Ethics". Our faculty has horizontal and vertical integration in medical education and our medical education is accredited.*
	Infrastructure	− Technological sufficiency − Using modern teaching techniques − Using simulation	5	*N5_Our infrastructural facilities are quite sufficient for the first 3 years.*
What are **the aspects** of ethics education at your school that need **to be improved**?	Academic staff’s quantity and quality	− Training and employing experts − Opening new departments − Strengthening existing departments	34	*N25_Our history of medicine and ethics department urgently needs a faculty member. Since the workforce trained in this field in our country is insufficient, this number needs to be increased as soon as possible.* *N51_Firstly, a history of medicine and ethics department should be established and a faculty member should be appointed.* *N58_The biggest deficiency in the field of ethics is the lack of sufficient workforce. Sustainability of our activities in the context of undergraduate, postgraduate and continuing medical education can be possible with the continuous provision of qualified workforce.* *N59_ [Not being able to] increase the number of academicians, especially young academics, is an important problem.*
	Education model	− Being up-to-date − Need for support and improvement	9	*N58_From time to time, our education materials should be changed and updated in line with the world examples of education models and by taking our social needs into consideration.*
	Vertical/clinical integration	− Formal ethics education in clinical years	12	*N73_[We need an] education program structured according to the system-based integration model that aims to ensure full integration of ethics education in the 3rd year and in clinical internships.*
	Infrastructure	-	6	*N66_...education model and infrastructure facilities need to be improved.*
	Assessment and evaluation methods	− Diversifying assessment and evaluation	1	*N17_I think that it is not enough to use only multiple choice test method as an assessment tool in ethics education. Student/team presentations in lessons; observational/interactive ethical dilemma discussions carried out with real patients/in hospital environments are also required.*

## Results

The results are presented according to the following categories: 1) study population; 2) demographic information about participating medical schools; 3) distribution of faculty members/instructors teaching ethics; 4) existence of research centers, and master’s and doctorate programs; 5) ethics courses in undergraduate medical education and departments/faculty members responsible for conducting them; 6) school years when ethics courses are taught; 7) teaching and learning methodologies used; and 8) assessment and evaluation.

### Study population

There were 101 universities with a medical education program in Turkey when the data were collected and all of them were taken as the sample. Twenty-six of them were foundation universities^2^. The Association for Evaluation and Accreditation of Medical Education Programs in Turkey^3^ accredited the medical education programs of 24 universities (30.4%).^4^ The average quota for student recruitment of all participating medical schools is 147.58 (min: 11; max: 391).

### Demographic information about the participating medical schools

Seventy-nine medical schools were represented in this study (response rate: 78%). Twenty medical schools (25.3%) were associated with foundation universities. Fifteen public and 17 foundation universities with a medical education program (total=32, 40%) were situated in the first six major cities (Istanbul, Ankara, Izmir, Bursa, Adana, and Antalya). While 91.1% of participating medical schools (*n*=72) had undergraduate ethics curricula, 30.4% (*n*=24) indicated that they did not have a DHME associated with their institutions. All medical schools in the aforementioned cities had undergraduate ethics curricula, and 90.6% of them (*n*=26) had a DHME. For the medical schools in other cities (*n*=47), these percentages are respectively as follows: 85.1% (*n*=40; *p*=0.04) and 55.3% (*n*=26). At 32.2% of public universities (*n*=19), and at 25% of foundation universities (*n*=5), there were no DHMEs associated with medical schools (*n*=0.74; Table 2).

**Table 2:** Numeric distribution of universities with a medical education program according to their statuses, and whether a department of history of medicine and ethics (DHME) is affiliated with them

Department of History of Medicine and Ethics (DHME)	Foundation University		Public University	
	n	%	n	%
Existent	15	75.0	40	67.8
Nonexistent	5	25.0	19	32.2
Total	20	100	59	100

*p*=0.74

The average number of medical students^5^ in a school year per instructor was 55.74 at foundation schools and 133.63 at public universities (*p*<0.001). Here, only faculty members working in DHMEs were considered. All schools with a DHME had undergraduate ethics curricula, while there were undergraduate ethics curricula at 70.8% (*n*=17) of the 24 schools without a DHME.

### Distribution of faculty members/instructors teaching ethics

There were no ethics faculty members or instructors at 27 medical schools (34.2%). Thirty-two medical schools (40.5%) had only one faculty member, while two faculty members were present at 11 schools (13.9%), and three to five at 9 schools (11.4%) (Table 3).

**Table 3:** Numeric distribution of faculty members/instructors responsible for teaching ethics at medical schools

Number of faculty members/instructors	Medical schools					
	Department of History of Medicine and Ethics (DHME)					
	Existent		Nonexistent		Subtotal	
	n	%	n	%	n	%
None	7	12.7	20	83.3	27	34.2
1	28	50.9	4	16.7	32	40.5
2	11	20.0	-	-	11	13.9
3	4	7.3	-	-	4	5.1
4	3	5.5	-	-	3	3.8
5	2	3.6	-	-	2	2.5
Total	55	100	24	100	79	100

Two schools had research assistants, but no senior faculty members or instructors involved in teaching ethics. Seven schools with a DHME had no faculty members or instructors. On the contrary, four schools without a DHME had a single faculty member each. Among the participants, 32 medical schools (40.5%) were situated in the six major cities. The number of teaching staff members at a medical school differs according to the city it is located in. At 93.8% of medical schools (*n*=30) in the major cities, DHMEs possess at least one faculty member; whereas among 47 schools in other cities, this percentage decreases to 53.2% (*n*=25). In other words, there were no faculty members at the remaining 22 medical schools located in smaller cities. While there was at least one professor in each of the DHMEs at 50% of the medical schools in major cities, this rate (departments with one professor) was only 19.1% for schools in other cities.

There were no bioethics or medical ethics professors at 68.4% of medical schools (*n*=54), and there were no associate professors at 84.8% of them (*n*=67). The percentage of those that had neither professors nor associate professors was 56.9 (*n*=45). Additionally, there were no professors at 54.5% (*n*=30), no associate professors at 76.4% (*n*=42), no assistant professors at 56.4% (*n*=31), and no instructors at 92.7% (*n*=51) of DHMEs. There was only one resident at 9.1% (*n*=5), two residents at 5.5% (*n*=3), and three residents at 1.8% (*n*=1) of them. This finding has been identified as an area for improvement through the answers given to the open-ended questions. The need to increase the quantity and quality of faculty members was emphasized by almost half of the participants. As a sub-theme, some mentioned that there was a need to train and employ qualified and competent teaching experts. Twenty-one medical schools cited that they had “experienced teaching staff” and that “faculty members from other disciplines participating in their lectures” was a strong aspect of their curricula. While 7 schools thought that the existing DHMEs “should be rendered functional and supported,” those without DHMEs expressed expectations that they would set them up urgently.

### Existence of research centers, and master’s and doctorate programs

**Fig. 1:**
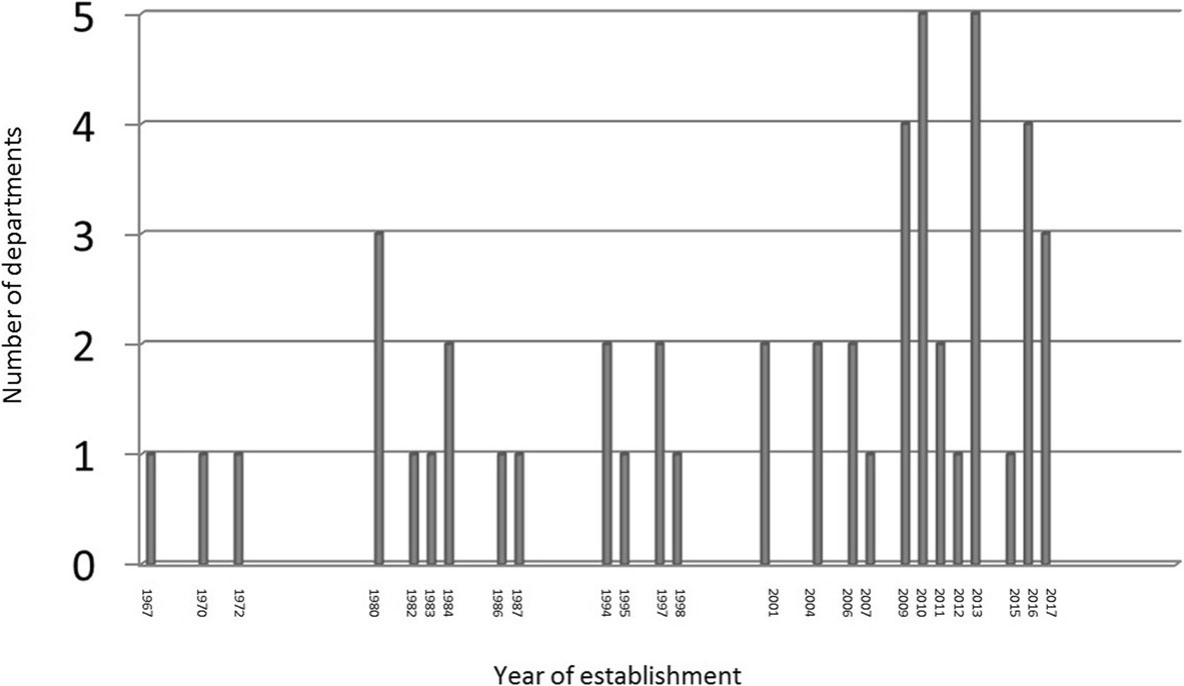
The number of DHMEs according to their year of establishment

Many DHMEs have been established in the last fifteen years in Turkey (Fig. 1).

Only two participants declared that there was a bioethics institute/bioethics research center associated with their institutions.

Eleven participants (13.9%) stated that they had master’s programs in bioethics/medical ethics, whereas 12 participants (15.2%) had doctoral programs in the history of medicine and ethics. At 10 universities, both master’s and doctoral programs in both fields were provided. Participants from two institutions reported that they only had doctoral programs. The majority of the master’s programs (81.8%, *n*=9) and doctoral programs (83%, *n*=10) were provided at universities situated in the five biggest cities (Istanbul, Ankara, Izmir, Bursa, and Adana). Our study did not investigate the content of these programs.

### Ethics courses in undergraduate medical education and departments/faculty members responsible for conducting them

The frequencies of the courses included in the undergraduate ethics curricula at participating medical schools are given in Table 4.

**Table 4:** The frequency of ethics courses according to the presence of DHMEs at medical schools

Ethics Courses*		Medical schools					
		Department of History of Medicine and Ethics (DHME)				Total (***n***=79)	
		Existent (***n***=55)		Nonexistent (***n***=24)			
**Main topics**	**Sub-topics**	**n (%)**	**M**	**n (%)**	**M**	**n (%)**	**M**
Four principles	Beneficence – Nonmaleficence	51 (92.7)	49.6 (90.2)	14 (58.3)	15 (62.5)	65 (82.3)	64,6 (81.8)
	Autonomy and individual responsibility	49 (89.1)		14 (58.3)		63 (79.7)	
	Consent	52 (94.5)		19 (79.2)		71 (89.9)	
	Persons without the capacity to consent	47 (85.5)		15 (62.5)		62 (78.5)	
	Equality, justice and equity	49 (89.1)		13 (54.2)		62 (78.5)	
Democratic rights and responsibilities	Human dignity and human rights	48 (87.3)	41.5 (75.4)	13 (54.2)	10.6 (44.4)	61 (77.2)	52.2 (66.0)
	Respect for vulnerable groups and personal integrity	43 (78.2)		11 (45.8)		54 (68.4)	
	Privacy and confidentiality	51 (92.7)		16 (66.7)		67 (84.8)	
	Non-discrimination and non-stigmatization	44 (80.0)		10 (41.7)		54 (68.4)	
	Respect for cultural diversity and pluralism	36 (65.5)		7 (29.2)		43 (54.4)	
	Gender	27 (49.1)		7 (29.2)		34 (43.0)	
Social rights and responsibilities	Solidarity and cooperation	35 (63.6)	37 (67.2)	7 (29.2)	8 (33.3)	42 (53.2)	45 (56.9)
	Social responsibility and health (social utility)	41 (74.5)		11 (45.8)		52 (65.8)	
	Sharing of benefits (Prioritization of patient’s beneficence against monopolization)	35 (63.6)		6 (25.0)		41 (51.9)	
Healthcare system and health policy	Right to health	52 (94.5)	42.2 (76.7)	16 (66.7)	11 (45.8)	68 (86.1)	53.2 (67.3)
	Justice in healthcare services	51 (92.7)		12 (50.0)		63 (79.7)	
	Resource allocation	42 (76.4)		7 (29.2)		49 (62.0)	
	Social determinants of health	33 (60.0)		8 (33.3)		41 (51.9)	
	Health policies	33 (60.0)		12 (50.0)		45 (57.0)	
Broader responsibilities	Protecting future generations	31 (56.4)	29 (52.7)	4 (16.7)	5 (20.8)	35 (44.3)	34 (43.0)
	Protection of the environment, the biosphere and biodiversity	27 (49.1)		6 (25.0)		33 (41.8)	
	Research integrity and publication ethics	46 (83.6)	46 (83.6)	15 (62.5)	15 (62.5)	61 (77.2)	61 (77.2)
	Health law	40 (72.7)	40 (72.7)	14 (58.3)	14 (58.3)	54 (68.4)	54 (68.4)
Overall mean values			40.8 (74.1)		11.2 (46.8)		52 (65.8)

*Eight participants did not answer this question. One participant stated that they did not have any medical students yet

Thirty-seven participants answered all the items about ethics course hours. The average number of theoretical course hours at these schools was 32.9 ±47.9 (min: 3; max: 290). Items left blank were interpreted to mean that the relevant courses were not taught at some schools. Only four participants responded to items asking about practical course hours. On the one hand, at two schools, there were no practical courses. On the other, another two stated that they had a total amount of 46 hours of practical sessions in the whole curriculum. One medical school mentioned that they had neither theoretical nor practical courses in ethics. Answers added to the “other” section by some participants were grouped as follows: ethics theories, physician-industry relationships, clinical ethics implementation, patient rights, duties and responsibilities of physicians, ethics committees, and international documents regarding ethics.

At some schools, ethics courses were conducted by faculty members working in departments other than DHMEs. At others, faculty members tenured in DHMEs could have another background besides bioethics or medical ethics.

One medical school representative remarked that the school did not have a department responsible for undergraduate ethics instruction. Ethics courses were taught only by DHMEs at 48.1% of the medical schools (*n*=38). At 11 schools (13.9%), ethics courses were taught by DHMEs in collaboration with other disciplines. Most of the ethics instruction at 49 schools (62%) was conducted by DHMEs, while other disciplines participated in some parts of the curricula. Yet, at 23 schools (29.1%) ethics courses were conducted only by departments other than DHMEs. For example, at 3.8% (*n*=3) of the medical schools Public Health departments gave ethics instruction, and at 5.1% of them (*n*=4) Forensic Medicine departments gave it. The distribution of the departments taking part in ethics curricula is presented in Table 5.

**Table 5:** Distribution of the departments taking part in ethics instruction

Departments taking part in ethics instruction	Medical schools	
	n	%
History of Medicine and Ethics	38	48.1
DHMEs in collaboration with other departments^a^	11	13.9
Other departments / disciplines	23	29.1
Not responded	7	8.9
Total	79	100

^a^: Public Health, Forensic Medicine, Family Medicine, Psychiatry, Physiology, General Surgery, Biochemistry, Biophysics, Neurosurgery, Anesthesia and Reanimation, Cardiovascular Surgery, Genetics, Anatomy, Pediatrics, Internal Diseases, Gynecology and Obstetrics, Pharmacology, Urology, Oncology, Endocrinology, Pulmonary Medicine, Histology, Otorhinolaryngology, Medical Education, and School of Nursery

Forty-two medical schools (53.2%) had at least one faculty member trained as an ethics expert or with a Ph.D. in medical ethics. Twenty-three participants replied to the open-ended question regarding faculty members who were responsible for ethics courses. Based on a content analysis of the answers, 7 medical schools cited that faculty members from clinical departments, as well as ethics scholars, contributed to ethics teaching as a strong aspect of their education programs. Twelve participants pointed out the need for instructors with different backgrounds as one of the aspects of their curricula that could be improved. All 23 participants thought that conducting undergraduate ethics education together with faculty members from a variety of disciplines was a crucial element for structuring the horizontal and vertical integration of ethics education into the overall medical curriculum.

### School years when ethics courses are taught

Participants gave more than one answer to the question of how many school years ethics education was given. Fifty-three schools (67%) taught ethics in the first year, 25 schools (31.6%) in the second, 50 schools (63.3%) in the third, 12 schools (15.2%) in the fourth, 17 schools (21.5%) in the fifth, and 14 schools (17.7%) in the sixth year. Forty-four participants (55.7%) specified that ethics was taught in more than two school years at their institutions, whereas at 28 medical schools, ethics courses were given for only one year. Table 6 shows the number of schools teaching ethics in different school years. It also shows how many schools teach ethics for only one year, during a combination of preclinical years, or preclinical and clinical years; and throughout the entire six years of medical education.

**Table 6:** Distribution of school years when ethics courses were given

Schools years that ethics courses are given	Medical schools	
	n	%
1^st^ year	12	15.1
2^nd^ year	3	3.8
3^rd^ year	10	12.7
5^th^ year	2	2.5
6^th^ year	1	1.3
Combinations of preclinical years	18	22.8
**Subtotal**	46	58.2
Combinations of preclinical and clinical years	24	30.4
All 6 years	2	2.5
None	7^a^	8.9
**Total**	79	100

^a^The number of participants who entered a response for this question.

In answers to the open-ended questions about the school years when ethics was taught, 7 participants emphasized that they practiced horizontal and vertical integration of ethics education during the clerkship and internship years at their institutions. Twelve participants mentioned that that this practice could be used to improve their curricula.

### Teaching and learning methodologies used

The methodologies showed diversity. Their distribution according to the institutions is shown in Table 7.

**Table 7:** Distribution of teaching and learning methods used in ethics education

Teaching and learning methods*	Medical schools	
	n	%
Classroom/auditorium seminar/lecture	70	88.6
Interactive presentation	39	49.4
Small group session	23	29.1
Case discussion	40	50.6
Discussion on movies/literary pieces	21	26.6
Problem based learning	10	12.7
Role-play with standardized patients	10	12.7
Practice with real patients	5	6.3

*More than one answer was given to this question

Twenty-four medical schools (30.4%) specified that they only used classroom seminars/lectures in ethics teaching, whereas one school (1.3%) used only interactive presentations. Fifty schools (63.3%) pointed out that they had been using more than one method. Thirty-two medical schools with a DHME (58.2%) and eight schools without a DHME (33.3%) stated that they used case analysis (*p*=0.04). Apart from that, there was no significant difference between the two groups of schools in terms of the teaching and learning methods used. Similarly, when medical schools were compared according the number of students per instructor, there was no significant difference between them.

There is no distinguished theme regarding teaching and learning methods in the answers to the open-ended questions. However, there are some mentions of education model, vertical and horizontal integration, number of students, and early encounters with ethics education. For example, the use of simulation, field studies, and student club activities were considered effective learning methods and stronger aspects of ethics curricula. As an area for improvement, some participants mentioned the necessity of updating their education model, including teaching and learning methods.

### Assessment and evaluation

The distribution of the methods used to assess and evaluate student attainment in ethics education is presented in Table 8.

**Table 8:** The distribution of assessment and evaluation methods used in ethics curricula

Assessment and evaluation methods	Medical schools	
	n	%
Multiple-choice test	68	86.1
Written exam	13	16.4
Case analysis	20	25.3
Homework/portfolio submission	17	21.5
360-degree evaluation	2	2.5
Role-play assessment	9	11.4
Oral exam	1	1.3

Forty participants (50.6%) stated that only multiple-choice tests were used at their institutions for assessing and evaluating education outcomes. Two medical schools (2.5%) used only written exams, and one utilized only case analysis. At 29 schools (36.7%), two or more assessment and evaluation methods were used. Nineteen medical schools with a DHME (34.5%) and only one school without a DHME stated that they used case analysis as an assessment and evaluation tool (*p*=0.003). Apart from that, there was no significant difference between these two groups of schools.

**Fig. 2:**
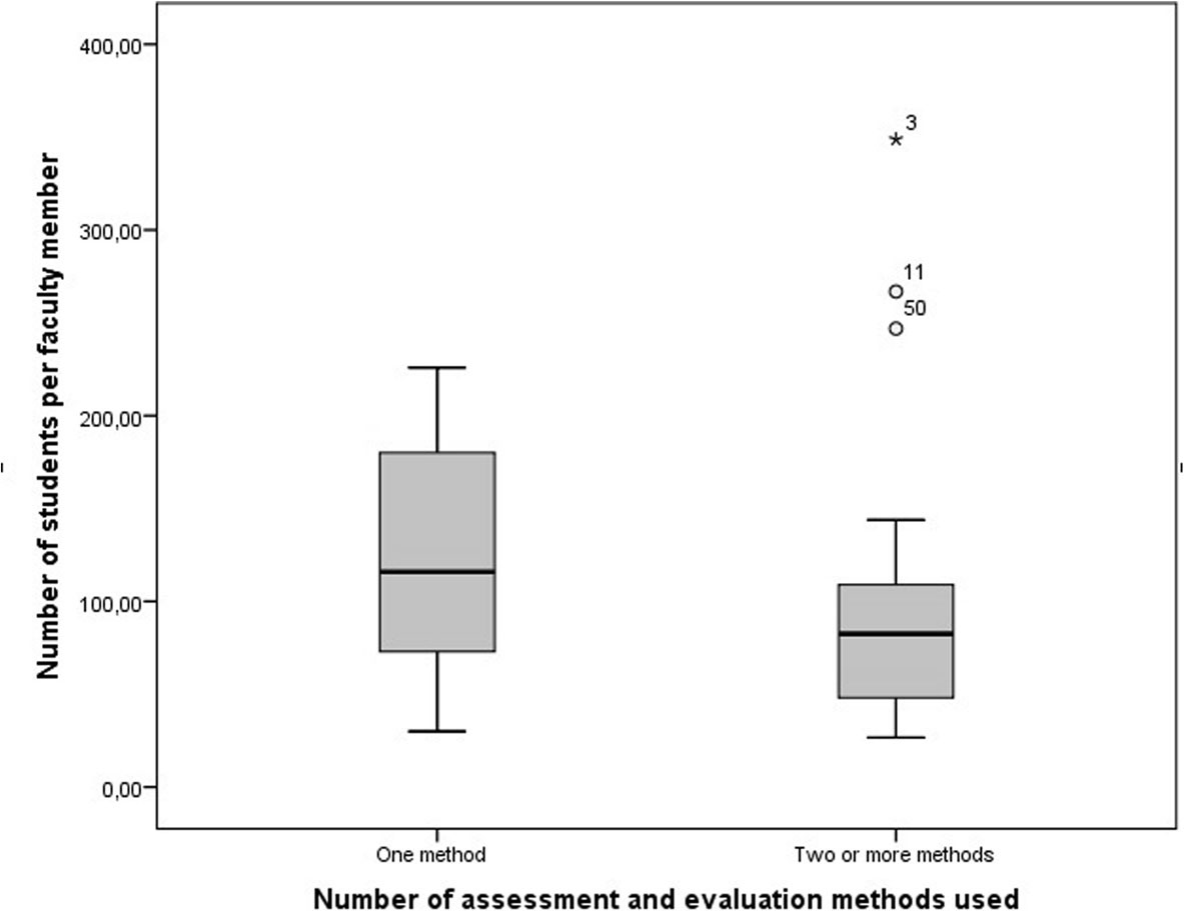
The number of assessment and evaluation methods used and the student quota per year for each school (*p*<0.05)

When the diversity of assessment and evaluation methods and the number of students per faculty member (who was responsible for ethics education) for each school are compared, it was found that the number of students per faculty member was fewer at schools using two or more methods than at those using only one method (p>0.05). While the average number of students per faculty member was 122.12 at schools where only one method was utilized, this score was 102.58 at faculties using multiple methods (Fig. 2). Having fewer students was pointed out as an advantage in the answers to the open-ended questions.

## Discussion

Recently, medical schools have been eager to provide medical ethics instruction and medical educators have been feeling responsible to ground medical ethics curricula in existing evidence of efficacy [12]. For this reason, the various attributes of ethics curricula have been a topic of interest for researchers in different countries. In their study, about the challenges of medical ethics education in training ethical doctors, Campbell et al. (2007) elucidated the view that medical education aims to produce reflective, empathetic, trustworthy doctors committed to patient welfare, who are able to adeptly deal with complexity and uncertainty in a given situation. They emphasize that medical ethics education should be multidisciplinary and multi-professional. It must be integrated horizontally and vertically into the medical curriculum so as to train knowledgeable professionals skilled to act in a clinically appropriate and efficient manner [38]. Besides, in a recent study on medical ethics curricula at medical schools in the UK, the US, and Australia, Giubilini et al. (2016) showed that the major challenges in teaching ethics were the negative effects of the hidden curriculum, how to apply ethical knowledge to real clinical cases, and how to shape the professional identity of future doctors through ethics education [13]. In this context, our research aimed to present countrywide cross-sectional knowledge for Turkey to ascertain where we are in terms of developing ethics curricula at medical schools and to discuss which aspects need improvement, and to what extent.

Given that the response rate was high (78%), some general inferences can be drawn. The overall findings suggest that ethics education is considered an integral part of medical education throughout the country. Ninety-one percent of respondents declared that there was a formal undergraduate ethics curriculum at their institutions. The cross analysis also showed that some sort of ethics education existed at schools without a curriculum as well, even though their content was unknown. The results highlight the following two main dimensions, which are discussed below: 1) substructure and workforce, and 2) ethics curricula.

### Substructure and workforce

Only two universities had bioethics centers that are designed to foster educational activities besides research and innovation. Almost one-third of participating medical schools did not have a DHME, and not all the official departments had experts in teaching ethics. There were neither professors nor assistant professors associated with DHMEs at more than 65% of the participating medical schools. This percentage increases when it comes to the number of associate professors and research assistants. There were no teaching assistants/instructors of ethics at participating schools. At almost one third of them, ethics education was given by scholars from a variety of departments other than history of medicine and ethics. Moreover, almost half of those who were responsible for ethics education did not have a Ph.D. in bioethics or medical ethics.

These findings suggest a deficiency in the number of teaching personnel who are responsible for developing, organizing, and/or conducting ethics education at a considerable number of medical schools in Turkey. Furthermore, there is an apparent imbalance in terms of the dissemination of the workforce among institutions. Based on the results, however, we cannot infer the existing teaching personnel’s competency levels.

Although most of the institutions appeared to have an undergraduate ethics curriculum, many lacked experts in ethics instruction. Academics with different backgrounds or from other disciplines taught ethics at those institutions. Eleven participants (14%) stated that more than one department, including the DHME, conducted ethics education at their schools. On the one hand, this may be favorable, especially considering that people with different backgrounds might share their own unique experiences of moral challenges and cases regarding their professional domains with students. On the other hand, this does not directly indicate that a multidisciplinary team organizes educational activities just because they are included in the teaching process. For instance, it is crucial that clinicians should take part in the organization, conduct, and evaluation of ethics education programs in cooperation with ethicists. This would lead to a constructive interdisciplinary learning environment and provide opportunities to supervise education that takes place outside the lecture halls [39]. Considering that asking for and receiving moral support from seniors is a learning moment, Cordingley et al. (2007) found that 80% of medical students received support from clinicians when they come across a morally distressing situation in their clinical years [40]. In another study conducted in Turkey, it was shown that half of the students (52%) thought ethicists should take part in ethics education, while others said all faculty members (21%); only clinicians (19%); or clinicians, academicians, or physicians with ethics teaching training (15%) should be in charge of ethics instruction. Thirty-one percent preferred more than one instructor from different disciplines [41]. Obviously, there is a demand for multidisciplinary ethics instruction. When there is discrepancy between what is taught in the classroom and actual experiences in the clinical setting, students need support from educators well-versed in ethics and professionalism to reconcile the mixed messages [42]. Nevertheless, the limited competency of those from other disciplines in terms of teaching ethics should be taken into consideration as an important drawback of this approach [39].

Additionally, the mean value of the number of students per instructor working at a DHME is considerably high, especially at public medical schools (*n*=133.63). The number of students admitted to medical schools has been increasing gradually every year. Moreover, new medical schools have kept emerging continuously for more than a decade in Turkey. The gap between the number of medical students who are in need of ethics education and the number of staff to provide them with it has been getting bigger. Most particularly, considering that only 15% of the participants declared that their institutions had bioethics and/or medical ethics master’s or Ph.D. programs, the official programs to train ethicists, the general need for a larger workforce in ethics instruction becomes even more obvious.

### Ethics curricula

Based on the results, we examine curricula in four segments: structure, content, teaching and learning approaches, and assessment and evaluation. We will not mention learning goals, as we did not include them in our study.

#### Structure

At Turkish medical schools, the first three years are spent in preclinical studies. Students start working in the clinic by the fourth year.

There was no formal ethics education at a small amount of schools (*n*=7). At others, ethics teaching mostly occurred in the preclinical years. Around 65% of respondents had classes in the first and/or the third years. The percentage of those that taught ethics in the second year was 31.6%. However, ethics was taught in one, two, or all the fourth, fifth, and sixth years (clinical years) at less than 20% of medical schools. At 43 schools, there was no formal program in the clinical years, while only 2 schools taught ethics in all 6 years. Our questionnaire was not developed to present differences, if any, in the degree and quality of the integration of ethics curricula into the overall professional education.

As has been well documented, medical students’ experiences and encounters in their clinical years are far more powerful in shaping their professional identities, as well as their moral sensitivity, moral attitudes, and competencies, than earlier training [43]. As a result, it is suggested that ethics education should be formally integrated into the whole 6-year medical curricula with a special concentration on the second half [1, 42]. It was also shown that things that are gained in practice and learned by doing, are more permanent than those obtained only by imagining or learning theoretically [44, 45]. Students welcome items covered by the former and add them to their professional toolbox to use when necessary, but they tend to forget elements of the latter, especially when they observe theory’s poor relevance to concrete incidents and relationships in their clinical years [13]. In today’s world, the disconnection between theory and real-world experiences leads to professional attrition and moral erosion in medical students through the internship period, and these effects intensify, especially towards graduation [46–49].

When considered in light of this framework, the findings point out a crucial deficiency in the organization and conduct of ethics education at most medical schools in Turkey. If there are zero, or inefficient, ethics education programs in the clinical years, then the moral component of medical students’ professionalization process is bound to be formally neglected. Ethics teachers would be deprived of opportunities to collaborate with their colleagues among clinicians, and that would limit their access to information regarding the moral climate of the healthcare setting. Their potential contribution to endeavors for improving clinical education might remain unrealized. Overall, they would not be able to prevent moral erosion among future physicians, let alone help them become competent professionals.

#### Content

As seen in Table 4, among the five main topics and two single courses, *Four principles* was included at most medical schools (81.8%). In addition, around two thirds of participants stated that they taught *Research integrity and publication ethics* (77.2%), *Health law* (68.4%), *Healthcare system and health policy* (67.3%), and *Democratic rights and responsibilities* (66%). *Social rights and responsibilities* (56.9%) and *Broader responsibilities for the environment and future generations* (43%) were the least popular courses. As eight participants did not respond to the question on instructional content, we can assume that they did not have any structured ethics curriculum. One medical school stated that it had no theoretical or practical ethics course. The topics we enquired about in this study were compatible with the core curriculum recommendations put forth by the UNESCO Division of Ethics of Science and Technology [5]. Overall, at many medical schools, most of the topics were covered in the curriculum. However, we lack reliable data regarding how these topics were taught to medical students, how much time was devoted to each, and which teaching methods were preferred. Only four schools responded to the item questioning the hours of practical sessions per topic. Therefore, we can infer that the content was mostly conveyed to students theoretically.

When we compared the data obtained from medical schools with a DHME and from those without it, we saw that the former group’s scores were considerably higher than those of the latter. For example, 90.2% of medical schools with a DHME taught topics related to the *Four principles*, while only 58.3% percent of others reported teaching them. The differences between the percentages for the rest of the topics present a similar pattern. On average, 74.1% of medical schools involving a DHME covered the courses on the list, whereas only 46.8% of the rest taught them. As seen here, for each of the main topics and single courses, there is a difference of at least 20 points between the percentage scores of the two groups of medical schools. This picture indicates the positive effect of the existence of a dedicated, official unit, such as a DHME, at a medical school for ethics education. Medical schools with a relevant department had more comprehensive education content mostly, though not fully, in accordance with the courses listed in contemporary recommendations for core curriculum. We can infer that they were more concerned about following the broadly accepted ethics issues. We can also conclude that this is because if schools have relevant departments/units, they are more likely to assign experts or scholars who could specifically focus on developing ethics education programs, training ethics teachers, and on compiling sources of information and knowledge under the same roof. On the contrary, medical schools without a DHME might be conducting ethics education solely with the support of experts from other fields, which is likely to be perceived as a side task both by the institution’s administration and by the faculty members who are in charge of teaching ethics.

#### Teaching and learning approaches

The results demonstrate that classroom lectures/seminars comprised a considerably higher portion of all educational methods used in ethics teaching in Turkey. Around 90% of participating schools had classroom lectures. It is the only method used at one-third of the schools (*n*=24), although many reported using more than one method in their programs. Forty participants reported using case discussions; however, we have no clue how cases were discussed with students— for example, as a part of a classroom lecture or separately in an interactive small-group session. Eighty-one percent of respondents used 1 to 4 methods in their curricula, which were classroom lectures/seminars, interactive presentations, small-group studies, and case discussions. Only 18.7% of participants used one or more of the following: narrative methods, problem-based learning sessions, role-playing, and virtual patients. As is known, these methods are distinguished from those previously mentioned because they are interactive and/or practice-based. Nevertheless, at most medical schools (*n*=70) classroom lectures/seminars were the predominant or the only teaching method. Case discussions (*n*=40) and interactive presentations (*n*=39) follow them in terms of prevalence. These findings suggest that most education programs are based on conveying information to students. In other words, they are mostly conducted at the knowledge level, suitable for cognitive attainment.

Weiss Robert et al. found that clinically-focused and multidisciplinary expertise-oriented learning approaches are welcomed by medical students. Besides theoretical teaching, the “most poignant lessons of professionalism and ethics are those that are lived out, discussed, and made meaningful in clinical situations” ([50], p.179). We found that such approaches were seldom or never used at most of the medical schools in Turkey. Considering the objectives of professional ethics education, we can assume that those programs fall short in fostering critical thinking, ethical awareness, empathy, and clinical ethical competency in students [38].

#### Assessment and evaluation

Our results show that most medical schools (86.1%; *n*=68) used multiple-choice tests to assess and evaluate attainment. In fact, at 40 schools, they were the only assessment and evaluation method used in ethics curricula. Case analysis (25.3%), homework/portfolio submission (21.5%), and written exams (15.2%) were the second most-used methods, although compared to multiple-choice tests, they seem supplementary. Other methods, such as 360-degree evaluation and role-play assessment based on multi-dimensional assessment and/or evaluating possible behavioral changes in students, were utilized by considerably fewer schools. These findings are compatible with the fact that ethics teaching was mostly based on theoretical classroom lectures at Turkish medical schools. Teachers often use multiple-choice tests to assess the amount of knowledge retained by students. According to the contemporary approaches, certain types of questions may be useful to evaluate other aspects of student attainment, such as reasoning in a sample case; however, our survey did not include such nuances. Yet we can plausibly conclude that more than half of the medical schools tested students merely for having the relevant knowledge, and most of the rest only assessed and evaluated the understanding of one aspect of ethical competency. However, because ethics education should also aim to cultivate virtues in future physicians and shape their attitudes and behaviors, if not character, toward professionalism, methods structured to assess and evaluate cognitive attainment would not be adequate to comprehensively measure educational outcomes [13].

This study has several limitations. Firstly, the responses were solely based on individual declarations and the data were not triangulated by comparing them with the institutions’ formal documents and programs. That might cause a certain degree of bias. Nevertheless, it might have been inefficient to go through the programs of all medical schools one by one, as most of them presumably needed to be updated in compliance with the current implementations. Besides, as our primary goal was to understand and analyze the ongoing teaching activities, we considered that the formal documents could be inadequate to provide insight concerning on-site practices. Secondly, due to socio-political factors, the academic system in Turkey underwent rapid changes during data collection. For example, some institutions were closed, while others were transformed or restructured, and new ones were set up. We included only the data collected from currently existing medical schools in the study. Thirdly, obtaining data by using different techniques (online questionnaires or telephone interviews) might have caused a certain amount of bias, but to a very little degree since the researcher who did the telephone interviews basically acted as a detached facilitator. Finally, the analysis suggested that the respondents might have perceived some terms differently, which risked undermining the common ground on which the discussions were based. For example, nomenclature regarding educational methods or assessment and evaluation seemed to have been misunderstood by the responders at some institutions. The researchers think that such a bias should have a minimum adverse effect on an accurate interpretation of the results.

The results highlight that staff qualified to teach ethics and integrated ethics education conducted by multidisciplinary teams are needed at medical schools in Turkey. Considering the general state of ethics education programs, it may be claimed that most medical schools are far from cultivating virtues and professionalism in future professionals. Endeavors aiming for contemporary topics should be encouraged. Finally, the role and effects of ethics education in the clinical years should be further studied.

## Conclusions

In this study, we present a general map of ethics curricula at medical schools in Turkey. Our overall results suggest a considerable deficiency in staff qualified to teach ethics in most medical schools. Therefore, we think that a further assessment study would reveal various dimensions of the need for teaching personnel such as their background and the pedagogical skills required of them. The last point depends on the priorities of an institution in terms of the learning goals of their ethics curricula and the teaching and learning approaches they adopt.

Our results also suggest that Turkish medical schools lack multidisciplinary ethics teaching activities. Training bioethics and medical ethics scholars eager and able to work in a multidisciplinary and multi-professional way to incorporate ethics curricula into the 6-year professional education seems to be an urgent necessity.

The content of ethics curricula varies among institutions in Turkey. Medical schools with a DHME were more likely to diversify teaching topics. At the majority of medical schools most of the internationally accepted topics are taught. However, we affirm the importance of endeavors aiming for a more contemporary set of topics addressing the current problems arising from the transformation of the healthcare system, social movements, and technological advancements, and those related to future generations, environmental issues, and the like.

Our results suggest that most medical schools fall short of giving students the necessary education for developing ethical attitudes and implementing their knowledge. In order to overcome this problem, the role and effects of ethics education in the clinical years should be reconsidered, and it should eventually be integrated into the whole medical curriculum.

Although our study presents cross-sectional results, it is reasonable to assume that ethics education programs will continue to gain importance in medical schools in Turkey. We can also assume that there will be continuous changes in the organization and conduct of ethics education parallel to the rapid transformation of healthcare service provision and structural changes at universities. Therefore, we think that a platform for monitoring bioethics and medical ethics education in Turkey should be established. Such an organization might comprise ethics educators from medical schools all over the country who network to find solutions to existing problems and build shared wisdom.

Turkey has developed an authentic, turbulent and ongoing practice of modernization historically and sociologically, and is an important economical and regional actor. The results of this study point out accomplishments achieved and challenges faced in providing ethics education to the growing number of medical students. We believe that the general state of affairs presented here might be considered both familiar and interesting by readers from many countries having similar contexts.

Finally, although our study quantifies some features of ethics curricula at medical schools in Turkey, we still lack sufficient insight into the thoughts of educators, administrators, and students on the merits and demerits of their ongoing programs, and changes they would like to see. For this purpose, we recommend conducting a further qualitative study that might enable us to understand the ethics education given at medical schools in Turkey both from the top-down and bottom-up approach.

## Supplementary information

**Additional file 1:** Questionnaire for the inventory analysis of ethics curricula at medical schools in Turkey.

## Data Availability

The datasets generated and/or analysed during the current study are available in the OpenICPSR repository, [https://www.openicpsr.org/openicpsr/project/109621/version/V1/view].

## Abbreviations

UNESCO: United Nations Educational, Scientific and Cultural Organization
WMA: World Medical Association
DHME: Department of history of medicine and ethics

## Footnotes

1 According to the legislation of the Council of Higher Education in Turkey, ethics and the history of medicine are considered one discipline. This branch of study is called “history of medicine and ethics” and the official affiliation of scholars working in these fields are the “departments of history of medicine and ethics” (DHMEs). The term bioethics is not officially used as a name for the concerned branch.

2 In Turkey, such universities are not called “private universities” but “foundation universities”, because they are not legislatively associated with companies that sponsor the foundations that establish and administrate them.

3 Turkish accreditation agency for undergraduate medical education. The agency is a registered association of the Higher Education Quality Board and is recognized by the World Medical Education Federation. It aims to contribute to improving the quality of medical education on a national level via the activities of information provision, evaluation, and accreditation.

4 This datum was taken from the agency’s website. http://tepdad.org.tr/en – Accessed 11.02.2019

5 The student quota numbers were taken from the official website of the Council of Higher Education. These numbers came from 2017. https://yokatlas.yok.gov.tr/lisans-bolum.php?b=10206 – Accessed 11.02.2019
