# Supplementary material for: The state of ethics education at medical schools in Turkey: taking stock and looking forward
Source: BMC Med Educ. 2020 May 24;20:162. doi: 10.1186/s12909-020-02058-9 (PMC7245803; doi:10.1186/s12909-020-02058-9)
Supplement: Supplementary file 1 — Additional file 1: Questionnaire for the inventory analysis of ethics curricula at medical schools in Turkey. [file 12909_2020_2058_MOESM1_ESM.docx]

**Appendix:**

**Questionnaire for the inventory analysis of ethics curricula at medical schools in Turkey**

**PART 1**

1. Name of your University / Medical School:

. . .

1. Is there an ethics curriculum at undergraduate level at your school?


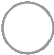
 Yes


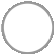
 No

1. Is there a department of the history of medicine and ethics at your school?


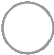
 Yes


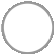
 No

1. If there is a department, which year was it founded?


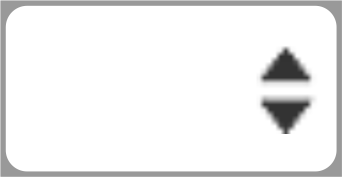


1. Is there a Bioethics Institute / Research Centre in your university?


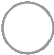
 Yes


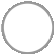
 No

1. If there is a Bioethics Institute / Research Centre, which year was it founded?


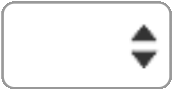


1. What is the number and what are the titles of the members of the department of history of medicine and ethics?

|  | None | 1 | 2 | 3 | 4 | 5 |
| --- | --- | --- | --- | --- | --- | --- |
| Professor |  |  |  |  |  |  |
| Assoc. Professor |  |  |  |  |  |  |
| Assist. Professor |  |  |  |  |  |  |
| Instructor |  |  |  |  |  |  |
| Specialist |  |  |  |  |  |  |
| Research/Teaching Assistant |  |  |  |  |  |  |
| Lecturer |  |  |  |  |  |  |

1. Is there an Ethics / Bioethics / Biomedical Ethics Master Programme in your university?


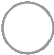
 Yes


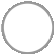
 No

1. Is there an Ethics / Bioethics / Biomedical Ethics Doctoral (PhD) Programme in your university?


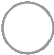
 Yes


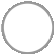
 No

**PART 2**

1. Please indicate the courses that exist in your undergraduate ethics curriculum:

|  | Existent | Nonexistent |
| --- | --- | --- |
| Human dignity and human rights |  |  |
| Beneficence – Non-maleficence |  |  |
| Autonomy and individual responsibility |  |  |
| Consent |  |  |
| Persons without the capacity to consent |  |  |
| Respect for vulnerable groups and personal integrity |  |  |
| Privacy and confidentiality |  |  |
| Equality, justice and equity |  |  |
| Non-discrimination and non-stigmatization |  |  |
| Respect for cultural diversity and pluralism |  |  |
| Solidarity and cooperation |  |  |
| Social responsibility and health (social utility) |  |  |
| Sharing of benefits (Prioritization of patient’s beneficence against monopolization) |  |  |
| Protecting future generations |  |  |
| Protection of the environment, the biosphere and biodiversity |  |  |
| Research integrity and publication ethics |  |  |
| Right to health |  |  |
| Justice in healthcare services |  |  |
| Resource allocation |  |  |
| Social determinants of health |  |  |
| Health policies |  |  |
| Gender |  |  |

Other (Please specify): . . .

1. Theoretical and practical hours of the courses:

|  | Theoretical | Practical |
| --- | --- | --- |
| Human dignity and human rights |  |  |
| Beneficence – Non-maleficence |  |  |
| Autonomy and individual responsibility |  |  |
| Consent |  |  |
| Persons without the capacity to consent |  |  |
| Respect for vulnerable groups and personal integrity |  |  |
| Privacy and confidentiality |  |  |
| Equality, justice and equity |  |  |
| Non-discrimination and non-stigmatization |  |  |
| Respect for cultural diversity and pluralism |  |  |
| Solidarity and cooperation |  |  |
| Social responsibility and health (social utility) |  |  |
| Sharing of benefits (Prioritization of patient’s beneficence against monopolization) |  |  |
| Protecting future generations |  |  |
| Protection of the environment, the biosphere and biodiversity |  |  |
| Research integrity and publication ethics |  |  |
| Right to health |  |  |
| Justice in healthcare services |  |  |
| Resource allocation |  |  |
| Social determinants of health |  |  |
| Health policies |  |  |
| Gender |  |  |
| Health law |  |  |

Other (Please specify): . . .

1. Please indicate the departments (disciplines) that teach ethics at your school:

🖵 History of Medicine and Ethics

🖵 Public Health

🖵 Forensic Medicine

🖵 Family Medicine

🖵 Psychiatry

🖵 Other (Please specify): . . . .

1. Do the ethics instructors have a Master’s/Doctorate degree in bioethics/medical ethics?


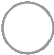
 Yes


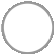
 No


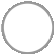
 Other (If there are instructors with different academic formation at your school, please indicate below.)

. . .

1. What year/grade is ethics taught at your school?

🖵 1

🖵 2

🖵 3

🖵 4

🖵 5

🖵 6

1. Teaching and learning methods used in ethics education:

🖵 Classroom lecture

🖵 Interactive presentation

🖵 Small-group work

🖵 Case discussion

🖵 Discussion on movies / literary works

🖵 PBL (Problem Based Learning)

🖵 Role-play with standardized patients

🖵 Practice with real patients

🖵 Other (Please specify): . . .

1. Assessment and evaluation methods used in ethics education:

🖵 Multiple-choice test

🖵 Written exam

🖵 Case analysis

🖵 Homework/portfolio submission

🖵 360 degree evaluation

🖵 Role-play evaluation

🖵 Other (Please specify): . . .

1. What are the **strong aspects** of the ethics education at your school? (Education model, infrastructural facilities, educational workforce, achieving the educational objectives etc.) Please write.
2. What are the **aspects** of ethics education at your school **that need to be improved**? (Education model, infrastructural facilities, educational workforce, achieving the educational objectives etc.) Please write.
